# T226. CLINICAL PREDICTORS OF FUNCTIONAL CAPACITY IN TREATMENT RESISTANT SCHIZOPHRENIA PATIENTS: COMPARISON WITH RESPONDER PATIENTS, ROLE OF NEGATIVE SYMPTOMS, PROBLEM SOLVING DYSFUNCTIONS, AND NEUROLOGICAL SOFT SIGNS

**DOI:** 10.1093/schbul/sby016.502

**Published:** 2018-04-01

**Authors:** Felice Iasevoli, Luigi D’Ambrosio, Danilo Notar Francesco, Eugenio Razzino, Camilla Avagliano, Elisabetta Filomena Buonaguro, Thomas Patterson, Andrea de Bartolomeis

**Affiliations:** 1 University of Naples “Federico II”; 2 University of California, San Diego

## Abstract

**Background:**

Treatment Resistant Schizophrenia (TRS) patients show more severe impairments in community functioning compared to Antipsychotic Responder Schizophrenia (ARS) patients. The scope of this work was to assess whether TRS patients suffer from more severe alterations in functional capacity, i.e. the baseline potential of a patient to function in the community, and whether factors affecting functional capacity differ between TRS and ARS patients.

**Methods:**

60 out of 182 eligible patients were included. A multistep diagnostic procedure to separate TRS from ARS was then used. Patients were administered a range of assessment tools including (but not limited to): the PANSS; cognitive performances tests; the Specific Level of Functioning (SLOF); the Neurological Evaluation Scale (NES); the UCSD Performance-Based Skills Assessment (UPSA) extended version. Univariate and multivariate statistics were performed. Significance was set at p<.05.

**Results:**

After controlling for covariates, no significant differences in both total and subscales UPSA scores were found between TRS and ARS patients. However, TRS patients constantly scored lower than ARS patients.

Stepwise regression was used to determine predictors of UPSA score. The first group encompassed clinical variables. In the whole sample, the final significant model, F(2,57)=18.848, p<.0005, adjusted R2=.37, included: PANSS negative subscale score and NES score. In TRS patients, the final significant model, F(3,24)=16.552, p<.0005, adjusted R2=.63, included PANSS negative scale score, education years, and NES score. In ARS patients, no significant models were found.

The second group included cognitive performance variables. In the whole sample, the final significant model, F(2,57)=7.64, p=.001, adjusted R2=.18, included Problem Solving and Verbal Memory. In TRS patients, the final significant model included Problem Solving and VisuoSpatial Memory. In ARS patients, the final significant model included Verbal Memory only.

The third group included psychosocial variables. In the whole sample, the final significant model, F(1,58)=18.82, p<.0005, adjusted R2=.23, included SLOF Area5 score only. In TRS patients the final significant model included SLOF Area1 score only, while in ARS patients, no significant models were found.

By hierarchical multiple regressions, NES score was found to be predictive of the highest UPSA score variance ratio among schizophrenia patients. The addition of PANSS Negative scale score and Problem Solving (in this order) led to a statistically significant increase in R2. No further models were found to add significant increase in R2. In TRS patients, PANSS Negative scale score was the variable that explained the most variance in UPSA score. The addition of Problem Solving and education years (in this order) led to a statistically significant increase in R2. No further models were found to add significant increase in R2, although NES score showed a trend toward significance.

At last, we performed a path analysis to evaluate the type (direct or indirect) and the direction of relationships among these variables and UPSA score. The only variables that were in direct relationship with UPSA score were PANSS Negative Scale score and Problem Solving. SLOF Area5 was in indirect relationship with UPSA score by PANSS Negative Scale score, while NES score relationship with UPSA score was mediated by Problem Solving.

**Discussion:**

Our study demonstrated that negative symptoms, altered cognitive performances, and more severe neurological soft signs were the major factors influencing functional capacity in schizophrenia patients. These factors were more relevant in TRS than in ARS patients.

